# ddPCR allows 16S rRNA gene amplicon sequencing of very small DNA amounts from low-biomass samples

**DOI:** 10.1186/s12866-021-02391-z

**Published:** 2021-12-18

**Authors:** Isabel Abellan-Schneyder, Andrea Janina Schusser, Klaus Neuhaus

**Affiliations:** Core Facility Microbiome, ZIEL – Institute for Food & Health, Technische Universität München, Freising, Germany

**Keywords:** ddPCR, Very small DNA amounts, Low-biomass samples, 16S rRNA gene sequencing

## Abstract

**Background:**

One limiting factor of short amplicon 16S rRNA gene sequencing approaches is the use of low DNA amounts in the amplicon generation step. Especially for low-biomass samples, insufficient or even commonly undetectable DNA amounts can limit or prohibit further analysis in standard protocols.

**Results:**

Using a newly established protocol, very low DNA input amounts were found sufficient for reliable detection of bacteria using 16S rRNA gene sequencing compared to standard protocols. The improved protocol includes an optimized amplification strategy by using a digital droplet PCR. We demonstrate how PCR products are generated even when using very low concentrated DNA, unable to be detected by using a Qubit. Importantly, the use of different 16S rRNA gene primers had a greater effect on the resulting taxonomical profiles compared to using high or very low initial DNA amounts.

**Conclusion:**

Our improved protocol takes advantage of ddPCR and allows faithful amplification of very low amounts of template. With this, samples of low bacterial biomass become comparable to those with high amounts of bacteria, since the first and most biasing steps are the same. Besides, it is imperative to state DNA concentrations and volumes used and to include negative controls indicating possible shifts in taxonomical profiles. Despite this, results produced by using different primer pairs cannot be easily compared.

**Supplementary Information:**

The online version contains supplementary material available at 10.1186/s12866-021-02391-z.

## Background

In 1985, the 16S rRNA gene was described for the first time as a molecular tool for identifying microbes that were previously shown to be unculturable [1]. This ubiquitous bacterial gene possesses special features containing conserved regions that enable primer binding and thus amplification, as well as hypervariable regions allowing phylogenetic differentiation. Thus, sequencing of the 16S rRNA gene still is the current method of choice to analyze taxonomical profiles of mixed bacterial communities [2, 3]. An often applied, easy, time- and cost-efficient method nowadays is short-amplicon sequencing using second-generation sequencers such as the Illumina MiSeq. Several factors affecting 16S rRNA gene sequencing results have been widely studied. Some of those are sampling and sample storage [4–7], the use of different variable regions or primers [8–12], sequence processing including the use of different denoising approaches, reference databases, and downstream analysis pipelines [13–18].

In addition to the above, it was previously shown that the extracted DNA can impact 16S rRNA analysis in two ways. Firstly, the use of different extraction methods or protocols influences the composition of a given sample [19–23]. More precisely, easy to lyse Gram-negative bacteria are favored by several extraction methods compared to hard to lyse Gram-positive bacteria [24–26]. Secondly, the DNA concentrations used for amplicon generation can influence the resulting taxonomical profiles [27]. This becomes even more critical when low biomass samples are analyzed, because contaminations (including eukaryotic non-target DNA) of those samples would more likely affect the resulting taxonomical profiles and, thus, could lead to distorted study results [28–30]. Lowering input amounts for 16S rRNA gene sequencing approaches are of particular interest for researchers investigating, e.g., the lower respiratory tract, preterm child microbiomes, stool samples of patients treated with antibiotics, milk samples, or any other sample type which is considered to be of low bacterial biomass [31–35]. Only very few studies tried to find minimum input amounts that are needed to produce reliable results. Brandt and Albertsen [36] defined a detection limit for bacteria in drinking water. They showed that if the bacterial input is 10^1^ cells/ml or smaller, several contaminating Operational Taxonomic Units (OTUs) appeared, and thus, sample outcomes could not be counted as reliable data. Multinu*,* et al. [27] reported that a minimum concentration of 40 pg/μl and an ideal concentration of > 200 pg/μl produce reliable 16S rRNA gene profiles of human stool samples. Velásquez-Mejía*,* et al. [37] showed that they needed at least 2 mg of fecal sample to extract sufficient DNA for 16S rRNA gene sequencing and the lowest successful amount of DNA used was 500 pg/μl in their study.

Here, we wanted to assess whether we could decrease the minimum input amount of DNA needed for reliable 16S rRNA gene sequencing of human stool and other samples even further. As a comparison, input amounts of 1–100 ng of DNA are commonly used for PCRs designated to perform later 16S rRNA gene sequencing. Illumina suggests using 12.5 ng total DNA input for a first step PCR (Illumina Inc., 16S Metagenomic Sequencing Library Preparation, Part #15044223 Rev.B). In our lab, we use 12 ng total DNA in our standard 16S rRNA gene sequencing approaches [38]. To enable the use of lower input amounts, we implemented a digital droplet PCR (ddPCR) approach followed by standard short amplicon 16S rRNA gene sequencing.

Common PCRs take place in larger reaction volumes between about 20 to 50 μl. An advancement is the ddPCR, which splits the larger volume into about 20,000 droplets, in which independent reactions occur within each droplet. Dividing the PCR volume into thousands of droplets has certain advantages: ddPCR was shown to be less sensitive to inhibitors [39] and was able to allow for selective and reproducible detection of rare alleles and the absolute quantification of targeted gene copy numbers [40]. Other benefits of ddPCR protocols are a reduced PCR bias by avoiding preference in the amplification of specific products over others by dividing the reaction mixture into small droplets, a simplified quantification compared to qPCR, and reduced consumable costs, as reaction volumes are small [41]. Gobert*,* et al. [42] showed a quantification method for low amounts of *Lactobacilli* in fecal samples using a ddPCR approach. There, quantification was possible even though only low numbers of the target strains were present within high background. Wouters*,* et al. [43] stated that by using a ddPCR protocol, they could detect very low amounts of a pathogen’s DNA in whole blood samples in as short as 4 h. In 2015, Boers*,* et al. [44] described a method were they performed micelle PCRs, a very similar concept to ddPCR, coupled with sequencing of the re-extracted 16S rRNA genes using a 454 GS Junior Sequencer platform (Roche). They showed that using the micelle PCR instead of a standard 16S PCR protocol reduces chimera formation in 16S rRNA profiling. Other studies investigating the coupling of microdroplet-based PCRs together with next generation sequencing strategies have been described previously [45–47], but for different purposes. In these studies, genes of interest, e.g., genes involved with congenital muscular dystrophies [45] or genes associated with diabetes and obesity [47] are screened by using a microdroplet-based PCR for enrichment next-generation sequencing reads for analysis. In the presented study, applicability and limits of ddPCR were tested in order to reliably obtain results for 16S rRNA gene sequencing with very low input amounts of initial DNA.

## Results

### Study overview

**Fig. 1:**
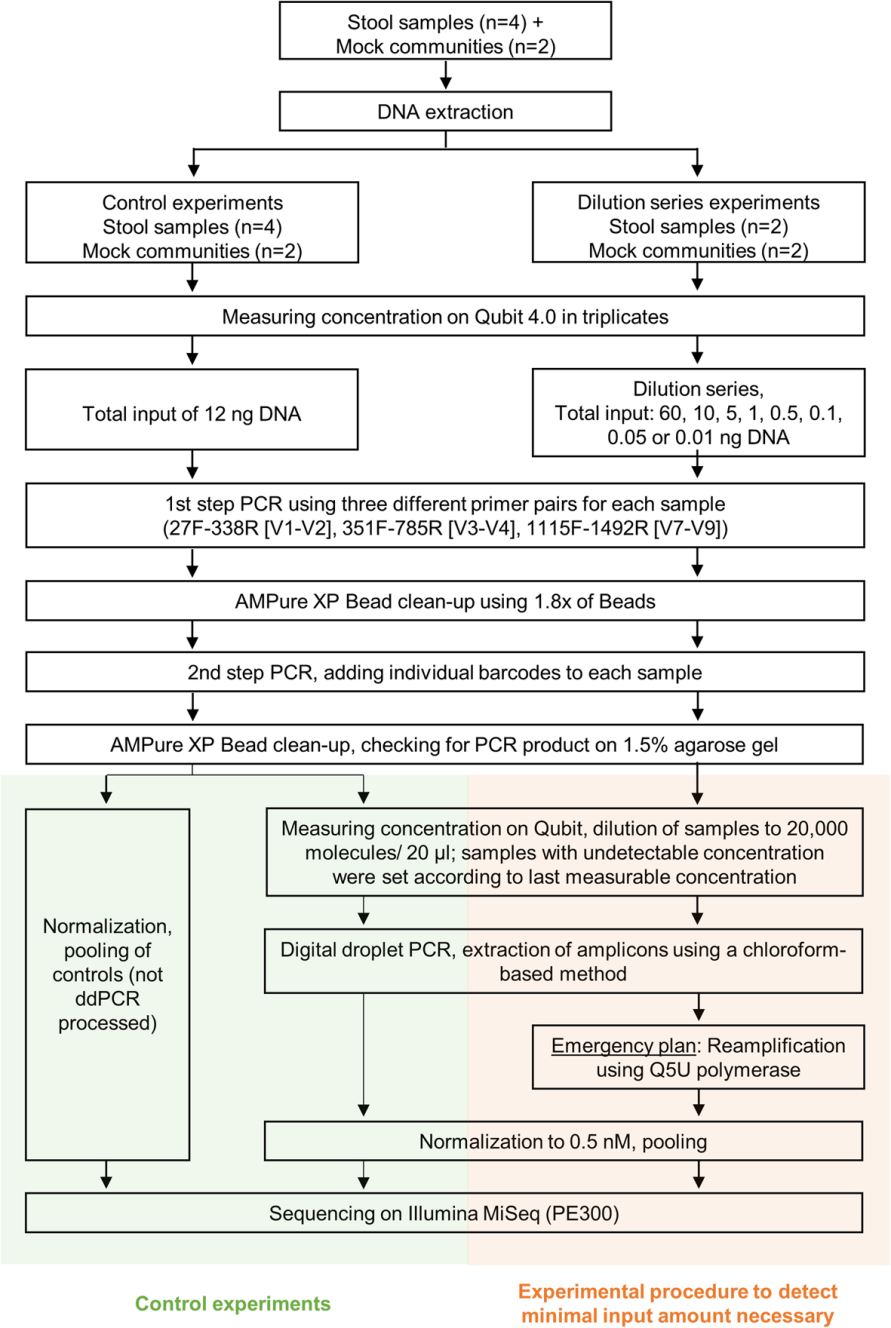
Overview of experimental procedure of this study**.** Experiments are divided into two parts. Left, control experiments (shaded green) were used for checking if the additional ddPCR step did not introduce bias in the resulting taxonomical profiles. Right, experimental procedures (shaded red) were described to detect the minimum input amount of DNA input necessary to produce reliable 16S rRNA gene sequencing results

The influence of the initial input amount of DNA was studied in detail. Towards this end, a general, published workflow for 16S rRNA gene amplicon sequencing was followed for the first part of the library preparation [38]. Subsequently, after purifying the amplicons after the 2nd-step PCR (i.e., barcoding), a ddPCR step was added allowing processing and later sequencing of initially very low DNA input amounts (Fig. 1).

First, to establish the new protocol, two different tests were performed. We prepared and sequenced, firstly, standard PCR products generated by using 12 ng DNA input (standard amount for 16S rRNA gene sequencing approaches in our laboratory). The very same samples were processed after the 2nd-step PCR by a further ddPCR step. Towards this end, samples were diluted, ddPCR performed and sequenced. By comparing the resulting sequences of both procedures, we evaluated whether the ddPCR step introduces bias.

Secondly, we performed dilution series and used decreasing amounts of initial DNA input (60, 10, 5, 1, 0.5, 0.1, 0.05, and 0.01 ng total input) in the 1st-step PCR. Subsequently, a standard 2nd-step PCR was performed, the resulting amplicon samples were diluted, processed by ddPCR, and sequenced. Using this approach, we evaluated whether we were able to produce reliable results even when DNA input amounts below 500 pg were used. Taken together, we compared resulting taxonomical profiles and whether they are independent of the DNA input amount used in 1st-step PCR or independent of an additional ddPCR (Fig. 1).

In brief, DNA of stool samples were extracted, concentrations were measured, dilution series were performed, and 1st-step PCRs were set up. In the 1st-step PCR, primer amplifying different V-regions were used (e.g., V1-V2, V3-V4, and V7-V9). Products were cleaned and used as a template for the 2nd-step PCR. Primers used include barcodes and the Illumina sequencing primer (P5 and P7). The resulting amplicons were again cleaned and it was checked whether the desired library size could be observed on agarose gels. The detection limit of the used GelRed dye is reported to be about 100 pg (Biotium, https://biotium.com/faqs/category/gelred-gelgreen/). However, it should be kept in mind that the actual limit depends on the used instrument’s capability and exposure settings. Sharp and conclusive bands were observable with our equipment and settings for at least 2–5 ng product loaded. For establishing the protocol, amplicons of the 2nd-step PCRs were diluted according to Formula (1), such that approximately one amplicon molecule is finally present per droplet. Next, ddPCR mixes were produced. The primers for the ddPCR used were plain P5 and P7 primers, which allow the re-amplification of the templates generated thus far. The final ddPCR amplicons were extracted and checked for adequate concentrations allowing 16S rRNA gene sequencing. If concentrations were still too low, which was the case for samples conducted using less than 50 pg initial DNA, the isolated amplicons were re-amplified in a standard PCR but using a Q5U polymerase with primer P5 and P7. This we referred to as “emergency plan”, since this additional step was able to “rescue” samples which otherwise would have failed in sequencing. In any case, all samples were sequenced on an Illumina MiSeq and compared.

### Determination of detection limits in standard 16S rRNA gene sequencing approaches

For all samples of the control experiment, products were detectable using agarose gels after the 2nd-step PCR. For the dilution series experiment, bands corresponding to the desired product were only visible for DNA inputs ≥5 ng total DNA, irrespective of which primers were used for amplification. After the ddPCR, bands could be observed on agarose gels for all samples amplified with primers targeting V7-V9. For V1-V2 samples, clear bands were visible for input amounts ≥50 pg. The detection limit for V3-V4 samples was higher; bands could only be detected for input amounts ≥500 pg for all samples, while some samples produced products at 100 pg already (Table 1).

**Table 1:**
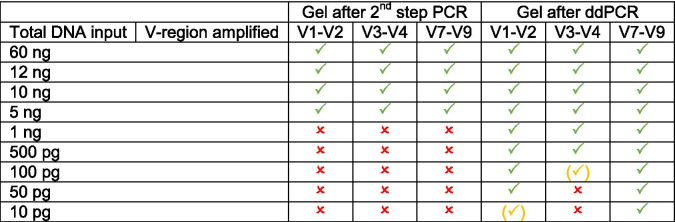
Visibility of bands corresponding to desired PCR products observed on 1.5% agarose gels. Green tick: bands were visible for all tested samples, yellow tick in brackets: bands were weak and/ or not visible for all tested samples. Red x: bands were not visible for none of the tested samples

If no band could be observed for a sample after ddPCR had been performed, re-amplification of the (invisible) product was conducted (marked as ‘emergency plan’ in Fig. 1). Importantly, re-amplification was only possible when an uracil-tolerant polymerase, e.g., *Taq* polymerase or a U-tolerant proofreading polymerase such as Q5U (NEB) or Phusion U Hot Start DNA Polymerase (Thermo Fisher) was used. The QX200 EvaGreen supermix contains some amounts of dUTP, causing ddPCR products to contain uracil subsequently. dUTP is used to allow the destruction of carry-over products from previous PCRs using Uracil-N-Glycosylase in the PCR mixture [48]. In any case, re-amplification with normal proof-reading polymerases such as the Phusion (Thermo Fisher) is inhibited and products can only be re-amplified with the mentioned U-tolerant polymerases.

The number of final sequenced reads, irrespectively of which approach was used, varied between 11,298 to 118,212 reads per sample with an average of 42,754 reads. The average read number of the negative controls was 506.

### Control experiment to assess the potential bias of the ddPCR step

In a first experimental setup, we assessed whether the integration of a ddPCR step after the 2nd-step PCR used for barcoding showed a bias on the β-diversity and the resulting taxonomical profiles of the samples. Ideally, samples originating from the same human donor, or the same mock community should not show any or only minor differences. We screened, therefore, four human samples (T1, T28, T29, T30) and two mock communities of known composition. The latter show different amounts of complexity as they are either composed of 8 different bacterial genera (Zymo mock community) or 18 different genera (ZIEL2 mock community). We further sequenced the samples using three different primer pairs amplifying V1-V2, V3-V4, and V7-V9.

**Fig. 2:**
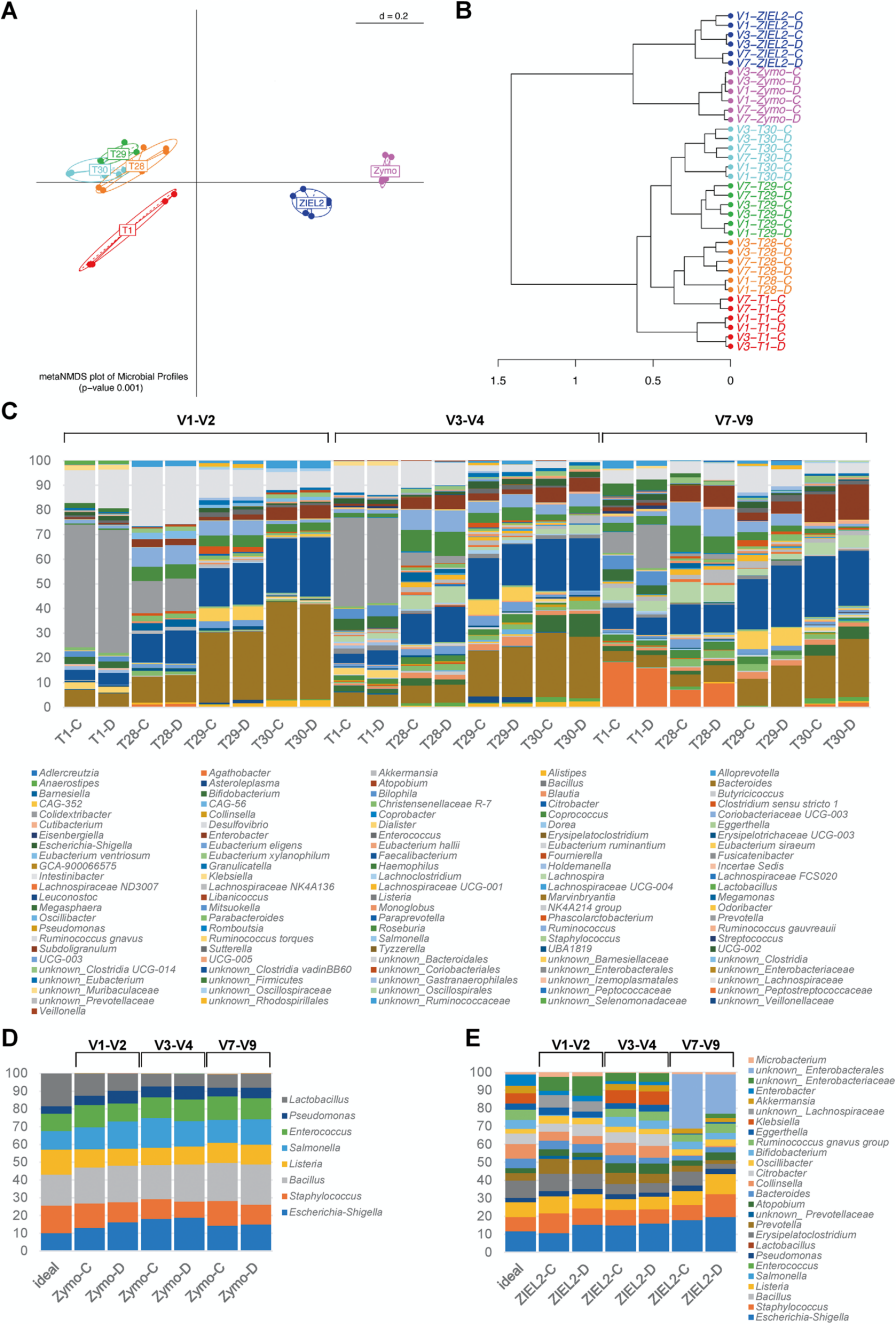
Control experiment to test for bias possibly introduced due to the extra ddPCR step after 2nd-step PCR. Samples processed with ddPCR (Samples marked “D” for ddPCR processed, 12 ng DNA used) are compared to standard short amplicon controls which were not ddPCR processed (Samples marked “C” for Control, 12 ng DNA used). Four human samples: T1 (red), T28 (orange), T29 (green), T30 (turquoise) and two mock communities: Zymo (pink), ZIEL2 (blue) were sequenced using primer pairs amplifying different V-regions. **A** Meta Multi-Dimensional Scaling (MDS) shows that samples cluster significantly differently due to their origin and not by preparation method. **B** The dendrogram shows that clustering is dependent on sample origin even though clustering within a sample is effected by the V-region targeted. **C** Taxonomic profiles at genus-level of Sample-C and Sample-D for human samples. **D** As before, for mock samples from Zymo and (**E**) and ZIEL2. Note, the taxonomic profiles at the genus-level show only minor differences when the same V-region is targeted

Comparing the results, we found that the differences introduced by using different primer pairs for the different V-regions caused profiles to be more distinct from each other than differences introduced by either the preparation method (standard protocol, marked as Sample-C in Fig. 2, vs. protocol with additional ddPCR, marked as Sample-D in Fig. 2). When clustering was performed on genus level, samples clustered by their origin (i.e., samples originating from the same donor cluster close to each other, even when amplified using different primer pairs). Concerning the latter, the difference between the tested samples (T1, T28, T29, T30, Zymo, and ZIEL2) was significant with a *p*-value of ≤0.001 tested with PERMANOVA (Fig. 2A). More importantly, we could demonstrate that the additional ddPCR had a smaller effect on the sample clustering than the use of different primer pairs (Fig. 2B). Further, the additional procressing step did not lead to significant differences in final sample composition as only minor shifts in the resulting taxonomical profiles of the samples were observed when control (samples-C) and ddPCR processed samples (samples-D) were compared (Fig. 2C). When comparing samples-C to samples-D in richness, we do not see a significant deviation in alpha-diversity. The resulting adjusted *p*-value found for the comparison of C-/D-samples was p_adj._ = 0.99. The Zymo mock community performed overall well (Fig. 2D), regardless of which V-region was targeted. For the more complex ZIEL2 mock community (Fig. 2E), we could show by calculating the generalized UniFrac distances against the ideal composition that the most accurate representation was produced by targeting the V3-V4 regions (see Supplementary Table 1).

### Estimation of minimal DNA input amounts for reliable 16S rRNA gene sequencing

In a second experimental set-up, dilution series of different total DNA input amounts were tested for the lowest DNA input possible producing reliable taxonomic profiles of human samples or mock communities. As before, samples were amplified using three different primer pairs. As shown in Table 1, detection limits varied for different V-regions. Thus, the taxonomic composition of each sample was checked for differences from either the actual composition in the case of mock communities or the taxonomical profiles achieved amplifying high initial DNA amounts in the case of the human samples. For the Zymo mock community, we found that Gram-negative bacterial genera such as *Escherichia*, *Pseudomonas,* or *Salmonella* were increasingly overestimated with descending DNA amounts used, while Gram-positive bacterial genera, e.g., *Lactobacillus*, *Listeria,* or *Staphylococcus* were progressively underestimated. When analyzing the ZIEL2 mock, primer-dependent issues became more prominent, which has been observed before [8]. In contrast to the Zymo mock community, no clear tendencies concerning different genera could be observed for the human samples despite the increase of spurious sequencing reads arising in very diluted samples (combined in “other”).

**Fig. 3:**
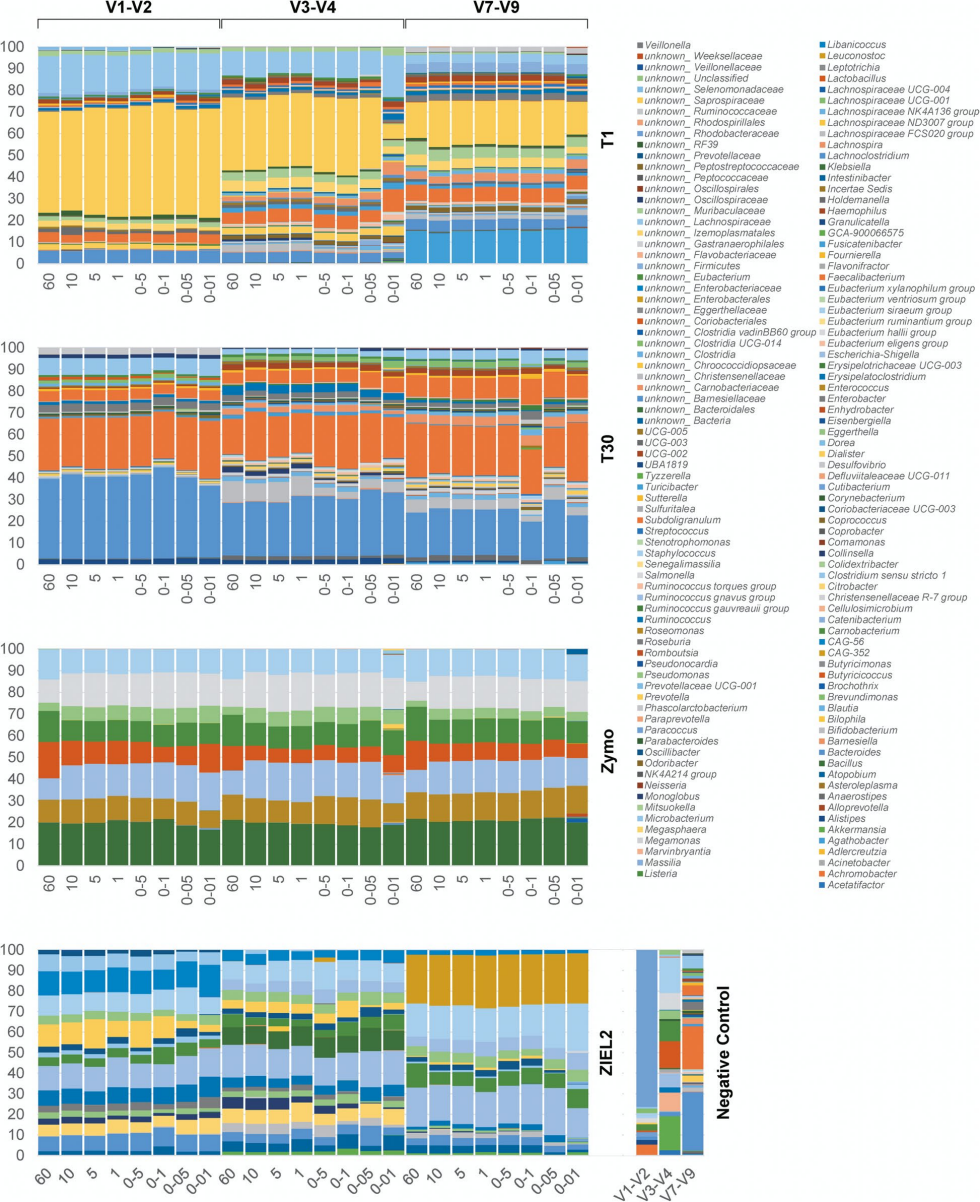
Taxonomical profiles at the genus level for two human samples (T1, T30) and two mock communities of known composition (Zymo, ZIEL2). For every sample, different initial DNA amounts were used for 1st-step PCRs and, further, different V-regions were targeted

For V3-V4 and V7-V9, deviations from the expected composition become more apparent with increasingly less DNA used as initial input (Fig. 3). For instance, amounts of 50 and 10 pg DNA did not always produced reliable results when taxonomical profiles at genus-level were analyzed. For the highly diluted Zymo mock, we observed increasing numbers of reads not representing members of the original mock community. Overall, for Zymo mock, the average amount of reads not corresponding to the expected bacteria was 0.9%. For V1-V2, the median amount of off-target reads for samples of 60 ng to 50 pg was 0.24% and for 10 pg input 1.27%. For V3-V4 and V7-V9, a drastically increased number of reads not matching the mock could be identified when using 10 pg input DNA. The average amount for off-target sequences was 0.19 and 0.24% for V3-V4 and V7-V9 respectively, of all reads concerning input amounts varying between 60 ng to 50 pg. The number of off-target reads for 10 pg samples reached 8.8 and 7.1% for V1-V2 and V7-V9, respectively. For human sample T1, 10 pg DNA input was not sufficient when targeting V3-V4. Deviations from the expected taxonomic profile are apparent for this low amount of input DNA used (Fig. 3). Thus, it seems that detection limits are not only V-region specific but also dependent on each sample. For instance, for T30 reliable profiles with just 10 pg input DNA targeting V3-V4 were produced, while T1 failed for the same combination and needed at least 50 pg input DNA. To verify reproducibility, we repeated the dilution series for T1, T30, and the Zymo mock community targeting V3-V4, which performed worst in the first experimental setup compared to V1-V2 and V7-V9. Only amplicons from using concentrations ≥100 pg produced reliable and repeatable results in the independent repetition (Supplementary Fig. 1).

### Proof of concept using water body samples

**Fig. 4:**
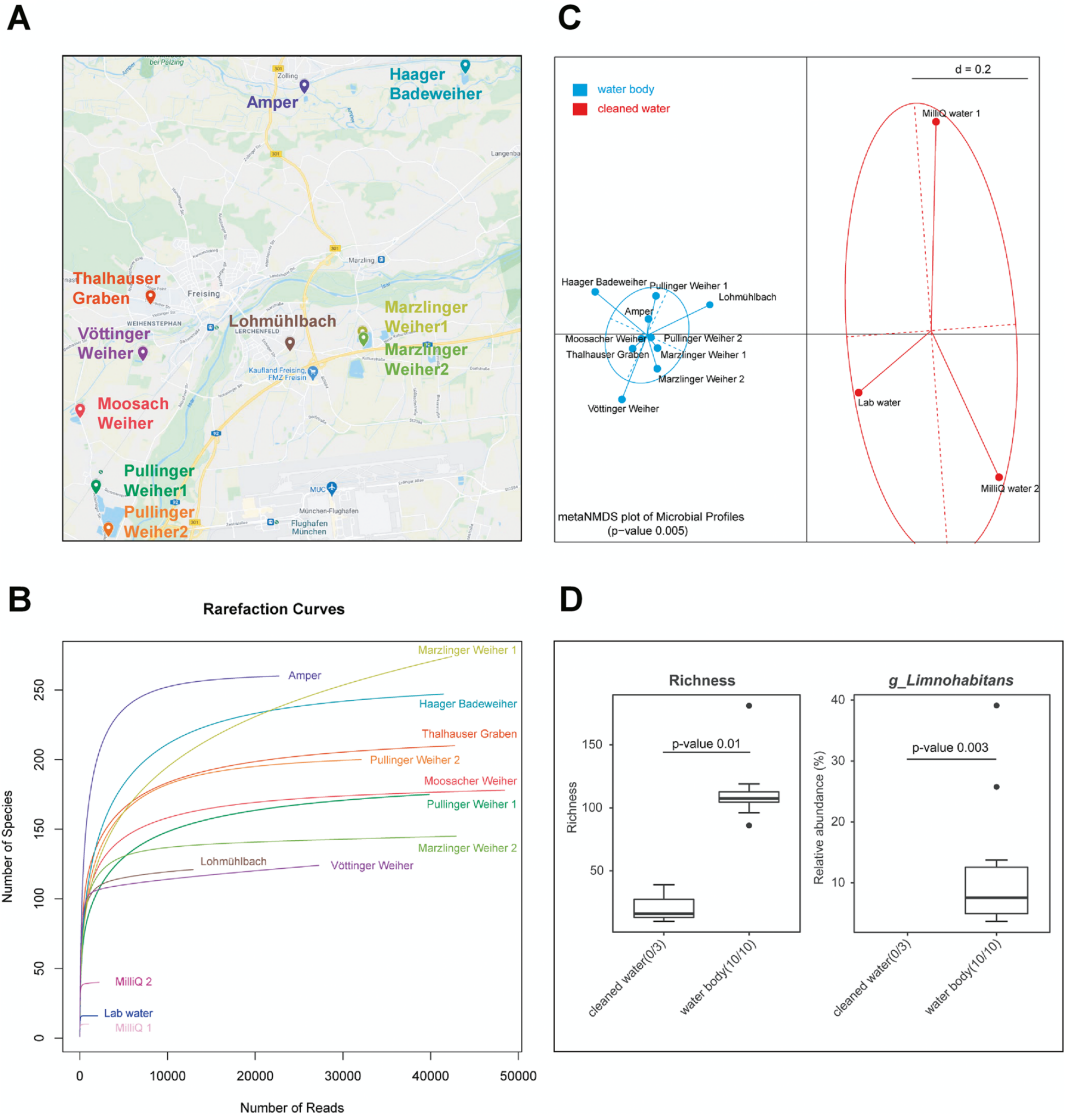
Proof of concept study for low biomass samples using water of ponds and rivers. **A** Ten different samples were selected from water bodies in and around the city of Freising, Germany. The map was provided by GoogleMaps. **B** The rarefaction curve shows that cleaned water samples (MilliQ and desalted water samples) have significantly lower number of reads and species compared to all other water body samples. **C** The multi-dimensional scaling (MDS) plot shows that cleaned water samples (‘control’) cluster significantly apart from the water body samples. **D** Significant differences were found when comparing the water body samples to the controls for richness or, e.g., the abundance of the genus *Limnohabitans*

To demonstrate that the proposed method is applicable to real low-biomass samples, we performed a proof-of-concept study using water body samples. Ten samples from different ponds and rivers near Freising, Germany (Fig. 4A) were collected. We analyzed the DNA of plain 15 ml water each. As controls, two MilliQ water samples and a desalted lab water sample were included accordingly and treated as the other water body samples.

At first, the standard 16S rRNA sequencing protocol was applied (1st-step PCR with 15x cycles and 2nd-step PCR with 10x cycles). The resulting amplicons were checked on an agarose gel for appearance of a band at around 600 bp of size. None of the water samples presented a visible band at the desired size. Moreover, after the purification with AMPure XP Beads, concentrations could only be determined for four out of the 13 samples and controls. Furthermore, the measured concentrations were with between 0.05 ng/μl to 0.09 ng/μl; thus, too low for the 16S rRNA gene sequencing. Next, we tested whether a simple re-amplification would result in sufficient products for all samples tested. Here, a standard P5-P7 PCR (15x cycles) was performed. However, even after re-amplification, no bands could be observed for any of the samples and neither for the controls. Resulting amplicon concentrations were still too low for most of the samples (data not shown). Therefore, we applied our proposed protocol. The 2nd-step PCR products were diluted and ddPCR processed. For all samples, except the controls (cleaned water samples: desalted lab water, MilliQ1 and MilliQ2) amplicons could be observed after the ddPCR. Concentrations ranged from 0.3 ng/μl to 4.2 ng/μl for the water body samples and 0.1 to 0.2 ng/μl for the controls. Sequencing was performed and the resulting analysis showed that we obtained for all water body samples useful results, which was not the case before. In all cases, the water body samples were more diverse compared to the control samples, which consisted of two MilliQ and a desalted lab water sample (Fig. 4B). For instance, all water body samples cluster significantly apart from the control samples in a metaNMDS plot (Fig. 4C). As expected, richness was significantly higher in any of the water body samples compared to the water controls. Further, we found that for example, *Limnohabitans* was present in all water body samples but not in the control samples (Fig. 4D). This bacterium is known to be in various numbers an important part for many freshwater habitats [49].

## Discussion

In this study it was investigated, how to obtain reliable 16S rRNA sequencing-based taxonomies even with very low input amounts of initial DNA. We found that the introduction of a ddPCR step after standard PCR-based library production allowed using low-DNA concentrated samples. The ddPCR enabled to successfully and reliably re-amplify 16S rRNA amplicons from the foregone PCR steps, even if they were not detectable in gel electrophoreses nor measurable using a Qubit. The minimal DNA input amounts successfully used in this study for samples processed were 50 pg DNA (equating to 1 pg/μl in the 1st-step PCR mix) for samples targeted using primers amplifying regions V1-V2 and V7-V9 and 100 pg DNA (equating to 5 pg/μl in the 1st-step PCR mix) for V3-V4. In contrast, Multinu*,* et al. [27] stated that the minimal concentrations of DNA input should be between 40 to 200 pg/μl. Further, Velásquez-Mejía*,* et al. [37] suggested to use at least 500 pg/μl DNA. Thus, we needed a maximum of at least 8-times less and often even lesser input amounts compared to these studies. Moreover, we could not confirm some of the other observations made by those groups. Multinu*,* et al. [27] described an overrepresentation of *Proteobacteria* and an underrepresentation of *Firmicutes* for low DNA input samples. When using ddPCR, no such general trend became obvious when analyzing human samples. For instance, for sample T1, the deviations between high and low DNA input were dependent on the targeted region, rather than on the amount of input DNA. When sample T1 was amplified using 27F and 338R primer (V1-V2), we could identify a reduction in *Firmicutes* and *Proteobacteria* for samples with lower DNA input amounts, whereas *Bacteroidetes* seemed to be overrepresented when using low DNA amounts. When V3-V4 was targeted in T1, *Firmicutes* and *Proteobacteria* seemed to be overrepresented and *Bacteroidetes* underrepresented. Concerning V7-V9, we saw no distinct change in *Firmicutes* or *Proteobacteria* amounts but an overrepresentation in *Bacteroidetes* for T1 (data are summarized in Supplementary Table 2). Even more, for T30, the trends do neither follow the shifts we saw for T1 nor those described by Multinu*,* et al. [27]. Generally, the phyla-level composition seems to be more inconsistent for T30 than for T1 when analyzing a dilution series within one targeted region (see Supplementary Table 3).

We conclude that the variation and shift in taxonomical compositions are mainly driven by using different primer pairs. This biasing factor was already intensively studied previously (e.g., [8, 9, 11, 50]). However, some further bias of under- or overrepresented taxa also is introduced due to distinct starting DNA concentrations. Here, we demonstrated that using ddPCR in case of limited input amounts (versus Control) had not a significant impact. Instead, samples cluster due to their origin, as wanted, but indeed also with the V-region(s) targeted, concurring with the above mentioned primer bias.

Human stool samples of healthy persons are not of low biomass. Nonetheless, we needed a sample type allowing using high and low DNA concentrations for comparison. The human stool samples should be only interpreted with some caution. We do not know the exact bacterial composition of these samples. However, in our proof of concept, we showed that the proposed method allows sequencing environmental samples, which are indeed of low biomass.

Interestingly, most of the commonly used protocols use high or even very high amounts of DNA in their protocols. To list only some examples: the Zymo Quick 16S NGS Library Prep Kit (Zymo Research Europe GmbH, Freiburg, Germany) aims for about 40 ng DNA that is free of PCR inhibitors; the QIAseq 16S kit (Qiagen, Hilden, Germany) recommends amounts of 12.5 ng, and the lowest amount usable is given with 1 ng, which is at least 20-fold higher compared to our protocol. Further, in the NEBNext Ultra DNA Library Prep kit (New England Biolabs, Ipswich, USA) for Illumina, 500 pg to 1 μg of input DNA is recommended. In this proof-of-principle study, we show that very-low initial DNA concentrations, which are by far lower than the recommended input amounts listed above, can be successfully sequenced, and reliably analyzed when implementing a ddPCR step. This is of special interest for low-bacterial biomass samples, such as milk, water samples, pathological or clinically relevant human samples including sputum, infant stool, biopsies, and others. While several 16S rRNA gene sequencing optimization protocols for such samples were already published (e.g., [31–35]), these studies aim at changing the parameters of existing protocols or try to reduce contamination sources in order to obtain taxonomic profiles of low-biomass samples. In contrast, ddPCR, which has to our knowledge not been applied to improve sequencing of low biomass samples, has only to be added at the end of a commonly used 16S rRNA gene sequencing protocol. While ddPCR methods were already described for quantification of microbial species or communities [39, 42, 51–53], to our knowledge, resulting PCR products were rarely re-extracted from the oil-aqueous suspensions for further use. Here we demonstrate that these products can be successfully sequenced, producing reliable taxonomic profiles. Even taking this a step further, these products can be re-amplified after ddPCR (e.g., in case of still too low concentrations), but uracil accepting polymerases must be used for following this “emergency plan”.

## Conclusions

Taken together, ddPCR, which splits the reaction volume in about 20,000 droplets, allows faithful amplification even of low amounts of template. Thus, sequencing of samples of low bacterial biomass (e.g., of a sick person with low bacterial loads), currently not easily accessible, can now be sequenced and compared with control samples of healthy persons with high amounts of bacteria. Besides, in order to improve comparability between publications it is important to always state the DNA concentrations and volumes used. Negative controls indicating possible shifts in taxonomical profiles are imperative. Finally, results produced by using different primer pairs cannot be easily compared.

## Methods

### Preparation of human gut samples

Stool samples were obtained from healthy volunteers of age after informed and written consent. An ethics approval is deemed unnecessary according to the statement given in the Drucksache 15/2849 of the German Bundestag about § 41 Abs. 2 Nr. 2 S. 1 and 2 Arzneimittelgesetz. Stool samples collected in stool sample tubes as described previously by Abellan-Schneyder*,* et al. [8].

### Extraction of DNA from stool samples

The DNA was isolated using a modified protocol by Godon*,* et al. [54] as described previously by Reitmeier*,* et al. [38] and Abellan-Schneyder*,* et al. [8].

### Extraction of DNA from mock communities

DNA of the Zymo mock community was purchased as a ready-to-use DNA mock (D6306, Zymo Research). The ZIEL2 mock community was prepared and extracted as described in Abellan-Schneyder*,* et al. [8]. In brief, every 19 bacterial strains (18 different bacterial genera) of diverse taxonomy were cultured and afterward harvested by centrifugation. Genomic DNA extraction was performed separately for each strain. For the ZIEL2 mock community DNA mixture, 12 ng of each bacterial DNA was pooled.

### Preparation of water samples

For each water body sample, at first 50 ml fresh water were collected in sterile falcon tubes and transported immediately to the lab. An ethanol precipitation was performed as previously described by Foote*,* et al. [55]. In brief, for each sample, 15 ml water were precipitated using 33 ml 100% EtOH and 1.5 ml 3 M NaOAc at − 80 °C for 2 h. Samples were centrifuged at 10,000×*g* for 10 min. The supernatant was discarded, the pellets were washed once with 80% EtOH and DNA extraction was performed using the same protocol as above. For the 1st-step PCR, 10 μl undiluted extracted DNA sample were used. The 1st- and 2nd-step PCR were performed as described below and PCR products were visualized after the 2nd-step PCR. The concentrations were determined on a Qubit 4.0 (Thermo Fisher) and dilutions were performed accordingly as described below.

### Determination of concentration and dilution of DNA input

Initial sample concentrations were measured in triplicates on a Qubit 4.0 (Thermo Fisher). According to the initial concentrations, stock solutions of 10 ng/μl and 1 ng/μl were set up and again measured in triplicates on a Qubit 4.0 (Thermo Fisher). The following dilution series, to reach the desired final concentrations (Table 2) were performed in 0.5 ml LoBinding Tubes (Eppendorf). After each dilution step, samples were briefly vortexed and spun down on a mini centrifuge.

**Table 2:** Concentrations and dilutions of DNA input used for 1st-step PCR reaction

Name	Total input DNA (ng)	Final DNA concentration in 50 μl PCR reaction (pg/μl)
60	60	1200
12	12	240
10	10	200
5	5	100
1	1	20
0.5	0.5	10
0.1	0.1	2
0.05	0.05	1
0.01	0.01	0.2

### Amplicon preparation

For amplification of the variable regions and addition of adapters, a 1st-step PCR was performed in 50 μl volume containing 10 μl DNA (total amounts are detailed in Table 1), 1× Phusion HF buffer, 0.2 mM dNTPs, 0.125 μM of each fw_primer and rv_primer, 7.5% DMSO and 0.25 μl of Phusion HF II DNA polymerase (Thermo Fisher). PCR was performed as followed: 98 °C for 40 s, followed by 15 cycles of 98 °C for 20 s, V-region specific annealing temperature (Table 3) for 40 s and 72 °C for 40 s, followed by a final extension step at 72 °C for 2 min. The structure of the primers was 5′ ➔ 3′: “overhang – [N]_15_ – 16S specific sequence” for the 1st-step and “P5/P7-8 bp Barcode – overhang” for the 2nd-step PCR. To enable multiplexing, barcodes were added in a 2nd-step PCR. Here, a 100 μl PCR was prepared using 10 μl of the 1st-step PCR product, 1× Phusion HF buffer, 0.2 mM dNTPs, 0.125 μM of each fw_barcode and rv_barcode primer, 0.25% DMSO, and 0.5 μl of Phusion HF II DNA polymerase. PCR conditions were 98 °C for 40 s, 10 cycles of 98 °C for 20 s, 55 °C for 40 s and 72 °C for 40 s as well as a final extension step at 72 °C for 2 min. Further details, e.g. work time estimations can be found in the work of Reitmeier*,* et al. [38].

**Table 3:** Variable region-specific forward and reverse primers and annealing temperature for 1st-step PCR

Region	Forward primer	Reverse primer	Annealing temperature	Reference
V1-V2	AGA GTT TGA TYM TGG CTC AG	GCT GCC TCC CGT AGG AGT	57 °C	Salter*,* et al. [28]
V3-V4	CCT ACG GGN GGC WGC AG	GAC TAC HVG GGT ATC TAA TCC	55 °C	Klindworth*,* et al. [10]
V7-V9	CAA CGA GCG CAA CCC T	GGT TAC CTT GTT ACG ACT T	51 °C	Turner*,* et al. [62]

### Library quality check

For validation and quality assurance, 8 μl of 2nd-step PCR product were loaded on a 1.5% agarose gel to perform gel electrophoresis. The remaining 92 μl of the 2nd-step PCR were purified with 0.6× AMPure XP beads. Concentrations of the 2nd-step PCR product were measured in triplicates using a Qubit 4.0.

### Digital droplet PCR

Amplicons generated in the two-step PCR (above) were amplified again using P5 and P7 primers in a ddPCR. At first, each sample was diluted to a concentration of approx. 20,000 copies/20 μl calculated by the Formula (1). The average library sizes were 486 bp for V1-V2, 602 bp for V3-V4, and 547 bp for V7-V9. Dilution series must be performed in LoBind tubes (Eppendorf) and in 1:10 steps to guarantee precise dilutions.

1 $$\frac{660\frac{\mathrm{g}}{\mathrm{mol}}\times \mathrm{average}\ \mathrm{l}\mathrm{ibrary}\ \mathrm{size}\ \left[\mathrm{bp}\right]}{\mathrm{6,022}\times {10}^{23}\ {\mathrm{mol}}^{-1}}\times {10}^9\times \frac{20000}{20\ \upmu \mathrm{l}}=\mathrm{concentration}\ \left[\frac{\mathrm{ng}}{\upmu \mathrm{l}}\right]$$

The composition of the reaction mixture for the ddPCR was as follows: 1× QX200™ EvaGreen® Supermix, 0.1 μM of P5 (forward) and 0.1 μM of P7 (reverse) primer, 2.5 μl DNA sample (1000 copies/μl) and water up to 25 μl. These ingredients were mixed thoroughly by vortexing and 20 μl of the mixture was transferred into a DG8™ Cartridge for the QX200 Droplet generator. Next, 70 μl of QX200 Droplet Generator Oil for EvaGreen was transferred into the oil well of the cartridge. Then a gasket was spanned over the cartridge and droplets were produced by the droplet generator following the Droplet Generator Instruction Manual (BioRad). Droplets were then transferred to a 96-well plate. Before starting the PCR in a thermocycler, the plate was sealed with a pierceable PCR Plate Heat Seal Foil (BioRad) using a PX1 PCR Plate Sealer (BioRad). PCR was performed in a PeqStar thermocycler (PeqLab) using cycling conditions as described in Table 4.

**Table 4:** Cycling conditions for ddPCR using P5 and P7 primer amplifying amplicons

Cycling step	Temperature (°C)	Time	Ramp rate	Number of cycles
Enzyme activation	95	5 min	2 °C/s	1
Denaturation	95	30 s		40
Annealing	59	1 min		
Extension	72	1 min		
Signal stabilization	4	5 min		1
	90	5 min		1
Hold	4	∞		1

The PCR products were recovered for further use of the amplicons. Each reaction was transferred into a clean 1.5 ml LoBind DNA tube (Eppendorf) and the lower oil phase (the specific oil is heavier than water) was discarded by pipetting. After adding 20 μL 1× TE buffer and 70 μl chloroform to the remaining aqueous phase, mixtures were vortexed for 1 min at high speed in a 2-ml adapter for the Vortex-Genie 2 (Thermo Fisher) and centrifuged at 15,500×g for 10 min. The upper aqueous phase (volume approx. 25 μl), containing amplicons, was separated by pipetting. Samples were purified using 1× AMPure XP beads and eluted in 20 μl H_2_O. The concentration was determined using the Qubit dsDNA HS Assay (Invitrogen). Analyzing the size of the ddPCR product, agarose gel electrophoresis (1.5%) was performed with 4 μl of each sample.

### Re-amplification of ddPCR using Q5U polymerase

If only unsufficient products for 16S rRNA gene sequencing could be extracted from the ddPCR, re-amplification was performed. Of note, re-amplification of the ddPCR product is only possible using a non-proofreading polymerase (e.g., *Taq* polymerase) or by using a polymerase that can read and amplify templates containing uracil (and inosine bases), e.g., Q5U or Phusion U DNA polymerase. The re-amplification reaction mix contained: 1× Q5U reaction buffer, 200 μM dNTPs (10 mM); 0.5 μM P5 primer (forward), 0.5 μM P7 primer (reverse), 5 μl ddPCR product (≤1 ng/μl), 0.02 U/μl Q5U Hot Start High-Fidelity DNA Polymerase, up to 50 μl nuclease-free H_2_O. Cycling conditions were set as described in Table 5.

**Table 5:** Cycling conditions for re-amplification of ddPCR

Cycling step	Temperature (°C)	Time	Number of cycles
Initial denaturation	98	30 s	1
Denaturation	95	10 s	5
Annealing	55	20 s	
Extension	72	45 s	
Final extension	90	5 min	1
Hold	4	∞	1

After the re-amplification, the PCR products were checked for the desired amplicon lengths via agarose gel electrophoresis. Samples showing bands at the desired size were purified by PAGE purification and eluted in 25 μl nuclease-free water. Concentrations were measured with the Qubit dsDNA HS Assay using 2 μl of the extracted amplicons.

### Sequencing

Samples were adjusted to 0.5 nM and pooled. Samples were sequenced in paired-end modus on a cartridge v3 using PE300 of a MiSeq system (Illumina, Inc.) following the manufacturer’s instructions and a final DNA concentration of 12 pM and 15% (v/v) PhiX standard library.

### Data analysis using IMNGS and Rhea

Data were processed using the Integrated Microbial Next-Generation Sequencing (IMNGS) pipeline [56], an in-house developed pipeline based on UPARSE [57]. In the advanced IMNGS options, allowed mismatches were set to one. Demultiplexing was performed using a minimum read-length of 250 bp and a maximum read-length of 600 bp. Forward trim was set to 30 bp and reverse trim length was 60 bp. The abundance filter was set to 0.0025 [58] and the filter of low-read samples was set to ‘off’. Chimeric reads were removed using UCHIME [59] and zero-radius operational taxonomic units (zOTUs) were produced using UNOISE 2 [60] and USEARCH v11.0.667. As reference database RDP (project release 11) was used. Refining of taxonomic data was performed using SILVA (release 138). Further analysis was performed in Rhea [61]. Rhea is a collection of R-scripts enabling comparison between samples. After normalization of data, *alpha*- and *beta*-diversities, richness, and other ecological parameters can be visualized. The script also performs taxonomic binning, enabling an insight on all known and unknown sequences of the microbial composition down to the genus level.

## Supplementary Information

**Additional file 1 **: **Supplementary Figure 1**. Multi-Dimensional Scaling (MDS) plots show that samples cluster significantly differently due to their origin (human donor and mock community). Moreover, timepoint 1 (e.g.,T30-1 samples) and timepoint 2 samples (e.g.,T30-2) cluster in proximity. (A) Clustering is performed only with samples prepared using ≥100 pg DNA input (lower limit for V3-V4). (B) All dilution samples were plotted.

**Additional file 2 **: **Supplementary Table 1.** Generalized UniFrac dissimilarity matrix for ZIEL2 samples in comparison to the ideal composition at the genus level. **Supplementary Table 2**. Phyla-level classification of dilution series for sample T1. In “other” the following phyla are combined*: Cyanobacteria*, *Desulfobacterota*, *Fusobacteriota*, *Verrucomicrobiota,* and unknown bacteria. **Supplementary Table 3**. Phyla-level classification of dilution series for sample T30. In “other” the following phyla are combined*: Cyanobacteria*, *Desulfobacterota*, *Fusobacteriota*, *Verrucomicrobiota,* and unknown bacteria.

## Data Availability

The datasets generated and analysed during the current study are available in the Sequence Read Archive repository, accession number PRJNA728558. https://www.ncbi.nlm.nih.gov/bioproject/PRJNA728558.

## Abbreviations

bp: Base pair
ddPCR: Digital droplet PCR
DMSO: Dimethyl sulfoxide
fw: Forward
IMNGS: Integrated Microbial Next-Generation Sequencing pipeline
OTU: Operational taxonomic units
rv: Reverse
V: Variable region of the 16S rRNA
v: Version
zOTU: Zero-radius OTU
