# Supplementary material for: ddPCR allows 16S rRNA gene amplicon sequencing of very small DNA amounts from low-biomass samples
Source: BMC Microbiol. 2021 Dec 18;21:349. doi: 10.1186/s12866-021-02391-z (PMC8684222; doi:10.1186/s12866-021-02391-z)
Supplement: Supplementary file 1 — Additional file 1 : Supplementary Figure 1. Multi-Dimensional Scaling (MDS) plots show that samples cluster significantly differently due to their origin (human donor and mock community). Moreover, timepoint 1 (e.g.,T30-1 samples) and timepoint 2 samples (e.g.,T30-2) cluster in proximity. (A) Clustering is performed only with samples prepared using ≥100 pg DNA input (lower limit for V3-V4). (B) All dilution samples were plotted. [file 12866_2021_2391_MOESM1_ESM.pdf]

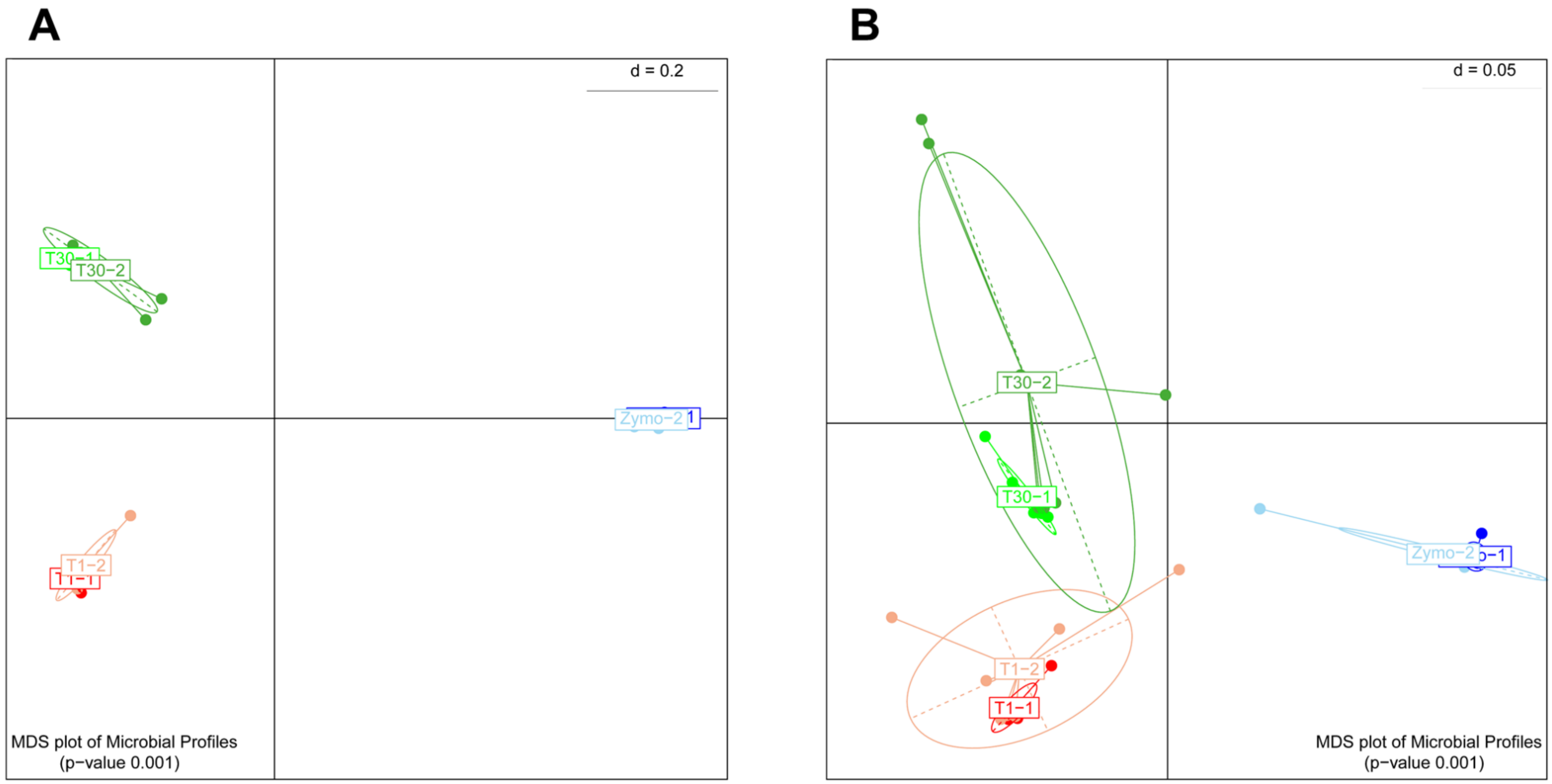

Supplementary Figure 1: Multi-Dimensional Scaling (MDS) plots show that samples cluster significantly differently due to their origin (human donor and mock community). Moreover, time point 1 (e.g., T30-1 samples) and time point 2 samples (e.g., T30-2) cluster in proximity. (A) Clustering is performed only with samples prepared using ≥100 pg DNA input (lower limit for V3-V4). (B) All dilution samples were plotted.
