# Supplementary material for: ddPCR allows 16S rRNA gene amplicon sequencing of very small DNA amounts from low-biomass samples
Source: BMC Microbiol. 2021 Dec 18;21:349. doi: 10.1186/s12866-021-02391-z (PMC8684222; doi:10.1186/s12866-021-02391-z)
Supplement: Supplementary file 2 — Additional file 2 : Supplementary Table 1. Generalized UniFrac dissimilarity matrix for ZIEL2 samples in comparison to the ideal composition at the genus level. Supplementary Table 2. Phyla-level classification of dilution series for sample T1. In “other” the following phyla are combined: Cyanobacteria, Desulfobacterota, Fusobacteriota, Verrucomicrobiota, and unknown bacteria. Supplementary Table 3. Phyla-level classification of dilution series for sample T30. In “other” the following phyla are combined: Cyanobacteria, Desulfobacterota, Fusobacteriota, Verrucomicrobiota, and unknown bacteria. [file 12866_2021_2391_MOESM2_ESM.pdf]

Supplementary tables

Supplementary Table 1: Generalized UniFrac dissimilarity matrix for ZIEL2 samples in comparison to the ideal composition at the genus level.

		ideal	V1-V2		V3-V4		V7-V9	
			ZIEL2-C	ZIEL2-D	ZIEL2-C	ZIEL2-D	ZIEL2-C	ZIEL2-D
ideal		0.00	0.88	0.88	0.39	0.38	1.00	1.00
V1-V2	ZIEL2-C	0.88	0.00	0.07	0.98	0.98	1.00	1.00
	ZIEL2-D	0.88	0.07	0.00	0.98	0.98	1.00	1.00
V3-V4	ZIEL2-C	0.39	0.98	0.98	0.00	0.04	1.00	1.00
	ZIEL2-D	0.38	0.98	0.98	0.04	0.00	1.00	1.00
V7-V9	ZIEL2-C	1.00	1.00	1.00	1.00	1.00	0.00	0.07
	ZIEL2-D	1.00	1.00	1.00	1.00	1.00	0.07	0.00

Supplementary Table 2: Phyla-level classification of dilution series for sample T1. In “other” the following phyla are combined*: Cyanobacteria*, *Desulfobacterota*, *Fusobacteriota*, *Verrucomicrobiota,* and unknown bacteria.

	T1-Samples, total gDNA input	*Actinobacteriota*	*Bacteroidota*	*Firmicutes*	*Proteobacteria*	other
V1-V2	60	0.11	57.17	41.54	1.13	0.05
	10	0.09	57.17	41.88	0.79	0.07
	5	0.14	60.20	38.77	0.83	0.06
	1	0.07	60.20	38.89	0.79	0.05
	0.5	0.09	61.61	37.32	0.91	0.08
	0.1	0.06	62.35	36.15	1.24	0.20
	0.05	0.01	58.50	40.98	0.45	0.06
	0.01	0.49	61.79	37.12	0.60	0.00
V3-V4	60	4.72	42.11	52.78	0.13	0.26
	10	4.77	42.12	52.84	0.04	0.23
	5	4.96	41.89	52.84	0.05	0.26
	1	4.74	42.77	51.99	0.24	0.26
	0.5	1.93	43.50	54.30	0.04	0.24
	0.1	1.67	43.91	54.26	0.03	0.13
	0.05	1.32	40.63	55.98	0.88	1.18
	0.01	1.83	13.68	79.76	3.71	1.01
V7-V9	60	2.65	23.82	72.46	0.95	0.12
	10	2.19	28.62	68.02	1.10	0.07
	5	2.10	27.41	69.50	0.89	0.10
	1	2.06	26.73	70.23	0.92	0.06
	0.5	1.93	27.26	69.83	0.91	0.08
	0.1	1.79	27.01	69.91	1.09	0.20
	0.05	1.66	27.36	69.94	0.83	0.22
	0.01	4.29	22.51	71.48	1.54	0.18

Supplementary Table 3: Phyla-level classification of dilution series for sample T30. In "other" the following phyla are combined: *Cyanobacteria*, *Desulfobacterota*, *Fusobacteriota*, *Verrucomicrobiota,* and unknown bacteria.

	T30-Samples, total gDNA input	*Actinobacteriota*	*Bacteroidota*	*Firmicutes*	*Proteobacteria*	other
V1-V2	60	1.88	41.32	55.32	1.14	0.33
	10	1.69	43.42	53.57	0.98	0.35
	5	1.84	42.24	54.71	0.90	0.32
	1	1.49	42.32	54.93	0.94	0.32
	0.5	1.45	43.14	54.13	1.01	0.27
	0.1	0.85	46.92	50.98	0.89	0.35
	0.05	0.92	40.98	54.88	2.64	0.58
	0.01	1.64	39.06	57.98	1.26	0.06
V3-V4	60	12.59	28.06	57.22	1.27	0.86
	10	10.23	29.01	58.97	1.14	0.65
	5	11.74	28.61	58.01	0.96	0.68
	1	11.27	31.66	55.37	1.09	0.62
	0.5	4.42	31.97	61.90	0.97	0.75
	0.1	3.77	30.31	64.08	1.15	0.68
	0.05	5.35	35.43	57.73	0.86	0.62
	0.01	6.34	33.56	58.13	0.89	1.08
V7-V9	60	6.58	22.18	69.69	1.13	0.43
	10	5.72	23.50	69.12	1.12	0.54
	5	5.28	23.18	70.05	1.02	0.47
	1	5.19	22.95	70.27	1.13	0.45
	0.5	5.26	23.39	69.94	0.93	0.48
	0.1	2.58	18.90	75.41	2.26	0.85
	0.05	6.07	29.13	62.43	1.99	0.38
	0.01	4.87	21.17	72.01	1.47	0.48
